# A favorable outcome in an infantile-onset Pompe patient with cross reactive immunological material (CRIM) negative disease with high dose enzyme replacement therapy and adjusted immunomodulation

**DOI:** 10.1016/j.ymgmr.2022.100893

**Published:** 2022-07-06

**Authors:** Shiri Curelaru, Ankit K. Desai, Daniel Fink, Yoav Zehavi, Priya S. Kishnani, Ronen Spiegel

**Affiliations:** a Department of Pediatrics B, Emek Medical Center, Afula, Israel; b Division of Medical Genetics, Department of Pediatrics, Duke University Health System, Durham, NC, United States; c Pediatric Cardiology Unit, Emek Medical Center, Afula, Israel; d Rappaport School of Medicine, Technion, Haifa, Israel

**Keywords:** Infantile-onset Pompe disease, Enzyme replacement therapy, Immunomodulation, Cross reactive immunological material (CRIM), *GAA* gene

## Abstract

Infantile onset Pompe disease (IOPD) is a rare devastating disease that presents in early infancy with rapidly progressive hypertrophic cardiomyopathy, severe generalized myopathy and death within the first year of life. The emergence of enzyme replacement therapy (ERT) with recombinant human acid alpha glucosidase (rhGAA) has improved the natural course of IOPD with a significant impact on cardiomyopathy but has a more limited effect on the progression of myopathy and consequently the later deterioration of the disease. Possible reasons for reduced ERT efficacy include insufficient enzyme, partial targeting of skeletal muscle and the development of IgG rhGAA antibodies especially in patients who are cross-reactive immunological material (CRIM) negative. We report a CRIM-negative IOPD female patient who started treatment upon diagnosis at 4.5 months with ERT at 20 mg/kg every other week and a course of combined immunomodulation with rituximab, methotrexate and IVIG according to the published Duke protocol and increased ERT within a month to 40 mg/kg/week. Despite initial good clinical response to ERT and immunomodulation, monthly monitoring identified a gradual increase of serum antibody titers to rhGAA necessitating a second course of immunomodulation with bortezomib and maintenance rituximab and methotrexate. A gradual reduction in frequency of immunotherapy was instituted and over a period of 14 months was discontinued. Serum anti-rhGAA antibody titers remained negative for 5 months since cessation of immunomodulation and the patient is now immune tolerant with recovery of CD19. At the age of 30 months the patient is walking independently and has normal cardiac function and anatomy. We recommend initiating ERT at 40 mg/kg/week in CRIM-negative IOPD patients, concomitant with immunomodulation and monthly monitoring of serum anti-rhGAA IgG titers upon confirmation of the diagnosis.

## 1 Introduction

Pompe disease (OMIM No. 232300, glycogen storage disease type II), an autosomal recessive, multisystemic neuromuscular disorder, is caused by biallelic mutations in the *GAA* gene encoding for the lysosomal enzyme acid alpha-glucosidase (GAA enzyme, EC 3.2.1.20) that degrades lysosomal glycogen [1]. In general, disease severity is associated with residual GAA enzyme activity. In classic infantile-onset Pompe disease (IOPD), GAA activity in skin fibroblasts and muscle is usually <1% in contrast with patients with late-onset Pompe disease (LOPD) [1]. Patients with IOPD present with, hypertrophic cardiomyopathy, failure to thrive, muscular hypotonia axial muscle weakness, and serum creatinine kinase (CK) elevation as early as the first days of life. IOPD is rapidly progressive, with the majority of untreated infants succumbing within the first year of life due to respiratory and cardiac failure with almost no motor milestones achieved [1,2]. In contrast, the presentation of LOPD is variable with the age of onset beyond infancy and without cardiac involvement, the disease course is usually consistent with progressive myopathy [1].

Intravenous enzyme replacement therapy (ERT) with recombinant human GAA (rhGAA; alglucosidase alfa), was approved in 2006 for treatment of Pompe disease. While the prognosis for patients with IOPD on ERT has generally improved, there remains substantial variability in the clinical responses [4]. IOPD patients are classified into two groups based on the presence/absence of endogenous GAA; patients who are unable to produce any GAA enzyme are designated cross-reactive immunological material (CRIM)-negative whereas those who are able to produce any GAA even if non-functional are designated CRIM-positive [3]. Published literature has shown that the development of high-sustained rhGAA IgG antibody titers (HSAT, defined as antibody titers ≥1:51,200 on two or more occasions at or beyond 6 months on ERT) is closely associated with a clinical decline in patients with IOPD [6]. Due to a complete absence of endogenous GAA, CRIM-negative patients are particularly at a higher risk for developing HSAT which can significantly impair the efficacy of ERT [6,7]. As such CRIM-negative status has been identified as a poor prognostic factor [6]. Other factors known to impact ERT efficacy include age at onset of treatment and the extent of pretreatment disease burden [4,5]. Accordingly, identification of IOPD within the first days of life due to newborn screening implementation improved outcomes of affected babies [8].

Over the past decade, it has become increasingly evident that IOPD patients who initially respond well to treatment continue to have sustained cardiac benefits but have residual myopathy and respiratory decline that progresses despite therapy typically noted after 20–24 months on a standard dose of ERT [3,9]. This is accompanied by a rise in serum CK and urinary glucose tetrasaccharide (Glc_4_). Urine Glc_4_ is a breakdown product of glycogen and increases in its levels suggests reduced glycogen clearance [10]. It has been shown that higher rhGAA dosing regimens have a beneficial effect on clinical outcomes in both patients with IOPD and early-onset LOPD who show either clinical plateau or decline when treated with a standard dose of ERT [[11], [12], [13], [14]].

In the following case study, we present a CRIM-negative IOPD patient, who had a favorable outcome by using a combination of high dose weekly ERT and an adjusted immunomodulation regimen to prevent the development of HSAT with monthly monitoring of rhGAA IgG antibody titers.

## 2 Case report

This 30-month-old female was born at 31 weeks of gestation due to early contractions and induced labor following an otherwise uneventful healthy bi-chorionic twin pregnancy. The parents are first-degree cousins of Arab Muslim descent. The patient developed respiratory distress syndrome requiring supplemental oxygen support until 5 weeks of age. She had normal brain ultrasound, echocardiogram, and electrocardiogram at one week of age.

At 4 months, she was admitted with fever, cough, dyspnea, and feeding difficulties. Vital signs were normal but oxygen saturation in room air was 90%. Physical examination revealed severe axial hypotonia with a significant head lag inability to raise her limbs against gravity and was unable to feed. A 3/6 systolic ejection type heart murmur was heard at the left sternal border without hepatosplenomegaly. She required supplemental oxygen and was fed by nasogastric tube. Arterial blood gases, serum ammonia, and lactate were normal, but she had elevated liver transaminases and CK levels. An echocardiogram found significant hypertrophic cardiomyopathy with left ventricular mass (LVM) of 60 g and left ventricular mass index (LVMI) of 221 g/m^2^ [upper normal limit: 64 g/m^2^] with severe left ventricular outflow obstruction. Propranolol therapy was initiated and IOPD disease was highly suspected. Genetic analysis of the *GAA* gene identified the homozygous c.2560C > T pathogenic variant resulting in premature truncation of the protein (p.Arg854Ter), which has been previously classified as a CRIM-negative IOPD variant [15]. In accordance, GAA enzyme activity level was 0 mmol/L/h [normal range > 2.0 mmol/L/h]. Her twin sister was also tested and found to be heterozygous with the same variant and had normal GAA enzyme activity [3.2 mmol/L/h].

**Fig. 1:**
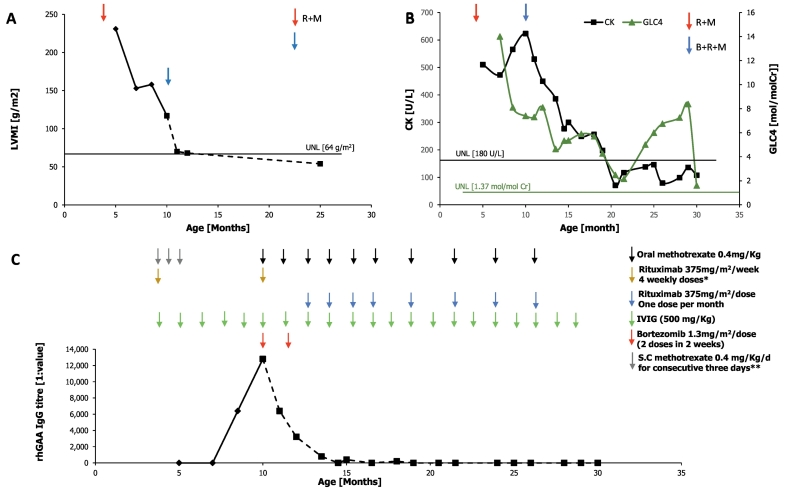
Immunomodulation protocol, anti rhGAA titer, cardiac mass and biomarker evolution over time. A. The course of left ventricular mass index (LVMI), B. The course of serum CK and urinary Glc_4_ in relation to ERT and immunomodulation treatment. C. Serum anti rhGAA antibody titers and detailed immunomodulation treatment according to patient's age. s.c = subcutaneous, R = rituximab, M = methotrexate, B = bortezomib. Glc_4_ = urinary glucose tetrasaccharide. *4 weekly doses of rituximab starting on the day before the first dose of ERT. **methotrexate given for three consecutive days (dose) starting on the day of first ERT and continuing with two additional doses at two weeks and four weeks after the first injection.

**Fig. 2:**
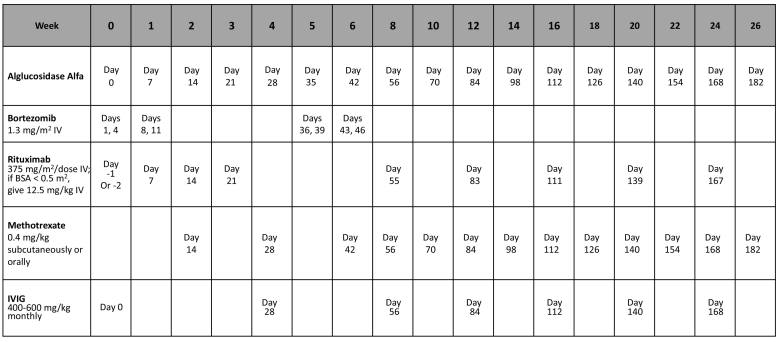
Immunomodulation protocol with bortezomib, rituximab, methotrexate and IVIG and maintenance therapy thereafter.

Upon diagnosis at age 4.5 months, the patient started ERT at 20 mg/kg every other week as well as a short immunosuppressive course according to the Duke protocol [18]. The protocol includes a short regimen (5 weeks) of rituximab, methotrexate and IVIG that are administered together with the first dose of ERT [18]. Given her severe CRIM-negative genotype she was switched to a higher dose ERT regimen of 40 mg/kg weekly within four weeks. She had a good clinical response with a gradual normalization of cardiac function and complete resolution of hypertrophy at 10 months of age (Fig. 1A) allowing discontinuation of beta blockers. Concomitantly, she displayed gradual improvement of motor abilities, began feeding well, and gained weight normally. To further increase the uptake of rhGAA by skeletal muscles, we added at age one year extended-release albuterol at a dose of 0.6 mg/kg/day. Given her CRIM-negative status and the high risk of developing neutralizing antibodies, rhGAA IgG antibody titers were monitored monthly. After 4 months on ERT (9 months of age), we noticed a rise in her rhGAA IgG antibody titer that reached a peak at 5.5 months of treatment [1:12,800] (Fig. 1C) while her CD19 count remained absent. Despite this increase the patient continued to display gross motor improvement, cardiac improvement and decrease in urinary Glc4 but elevation of serum CK levels (Fig. 1A,B). Aiming to improve her immune tolerability and decrease the development of neutralizing antibodies, bortezomib induction was initiated five months after ERT was started, as well as a further course of rituximab and methotrexate [16]. To support ongoing immune tolerance, she continued a monthly maintenance rituximab and methotrexate regimen as well as monthly IVIG infusions (Omr-Igg-am®) to provide passive immunity. The detailed protocol of second immunomodulation with bortezomib, rituximab methotrexate and IVIG including timing and rituximab maintenance thereafter is presented in Fig. 2. Accordingly, the patient had complete clearance of rhGAA IgG antibody at the age of 14 months (Fig. 1C) and continued gross motor improvement. She started to walk independently at 20 months, and her cardiology examinations were normal. Her CK levels which were raised above normal levels gradually decreased to normal levels at age 18 months and remained within normal range thereafter (Fig. 1B). Concomitantly, her urinary Glc_4_ levels remained stable although mildly increased above normal (Fig. 1B). Audiologic assessment showed mild conductive hearing impairment of 25–30 dB with no sensori-neural deficit.

Six months after bortezomib induction, antibody titer remained absent and therefore we spaced rituximab and methotrexate to once in two months and after additional six months were completely discontinued safely at age 26 months (Fig. 1C). In addition to monthly monitoring of serum rhGAA IgG antibody titer, we monitored CD19 counts (by flow cytometry) and serum immunoglobulin counts. Accordingly, the patient had significantly decreased CD19 (1–3% of total lymphocytes) and reduced total IgG counts and therefore continued to receive monthly IVIG at the dose of 500 mg/Kg. The patient has not developed any infusion-related reactions, but she had two episodes of line infections, both before induction with bortezomib, that were treated successfully with intravenous antibiotics and line replacement. Importantly, in spite of her immunocompromised state, she has not suffered any recurrent or opportunistic infections.

On her most recent visit, at 30 months (25 months on ERT), the patient is walking independently for >100 m, running although still clumsy, and able to climb stairs with assistance. She is eating independently, does not need feeding support and has no swallowing difficulties. She has no respiratory compromise and was able to tolerate well upper respiratory viral infections. Notably, the patient is already 4 months off immunomodulatory treatment and continues to maintain low antibody titers. Her current echocardiographic examination is normal with a normal LVMI of 54 g/m^2^ (Fig. 1A). The patient is receiving her weekly ERT via a Port-a Cath central line and in spite of the ongoing COVID-19 epidemic and the two episodes of line infection has not missed infusions. Importantly, absolute neutrophil count remained stably normal, her CD19 count completely recovered following discontinuation of rituximab and methotrexate at 27 months and her liver enzymes remained normal and serum total IgG has normalized allowing safe discontinuation of monthly IVIG at 28 months of age. Overall, she had no clinical signs of peripheral neuropathy.

## 3 Discussion

In this report, we present a favorable outcome in a CRIM-negative IOPD baby born prematurely at 31-weeks of gestation and initiated ERT and immunomodulation upon diagnosis at 4.5 months (corrected age 2.5 months). At 30 months of age, the patient has normal cardiac function, able to walk unaided, and has normal levels of skeletal muscle markers including serum CK and transaminases with mildly increased but stable urinary Glc4 levels confirming successful glycogen clearance [17]. Importantly, immunomodulatory regimen used in this patient was safe.

The CRIM-negative IOPD represents the most severe form of Pompe disease. In addition to the infantile-onset that includes significant life-threatening cardiac involvement; these patients also experience complications that result from immune response to ERT and the potential development of HSAT that negatively impact ERT efficacy. In our patient, upon the identification of the homozygous null CRIM-negative c.2560C > T variant, we started intravenous rhGAA at a standard dose of 20 mg/kg every other week after initiation of immunomodulatory therapy aiming to prevent the development of anti rhGAA neutralizing antibodies. Given the severe mutation and expected severe clinical course as well as the late onset of ERT, we quickly switched to a significantly higher dose of 40 mg/kg weekly without evidence of adverse events. Prior studies and case descriptions suggested that this high dose regimen (four times compared with the standard dose) may exert a more beneficial effect in certain LOPD and IOPD patients especially those with deterioration on standard ERT dosing [[11], [12], [13], [14]]. Overall, weekly infusions with high dose preparations were tolerated in our patient without infusion related reactions.

The negative impact of elevated levels of anti-rhGAA IgG antibody titers in CRIM-negative IOPD patients was previously demonstrated [3,7,9]. In our patient, despite initial good response to immunomodulation course according to the Duke's protocol (18), monthly monitoring revealed a gradual rise in the anti-rhGAA IgG antibody titers while the patient continued to make gross motor progress. A second bortezomib-based immune tolerance induction was safely tolerated and resulted in complete clearance of anti-rhGAA IgG antibody titers (Fig. 1C). Bortezomib, a proteasome inhibitor that targets short and long-lived plasma cells was previously shown to be both safe and effective in controlling Pompe disease patients who developed HSAT following ERT [16]. This time we continued with monthly maintenance of rituximab and methotrexate with later tapering down until complete cessation 16 months after bortezomib induction. Therefore, we suggest that CRIM-negative IOPD patients should receive immunomodulation with the onset of ERT, accompanied by monthly monitoring of serum anti rhGAA antibody titers. In cases where a progressive increase in anti rhGAA titers is observed especially when achieving a titer of 12,800 even if clinical improvement still continues, we suggest to initiate a short regimen with a plasma cell targeting agent, such as bortezomib associated with maintenance rituximab with gradual decrease until cessation to ensure long-term immune tolerance to prevent clinical deterioration. In general, our recommendation for rituximab and methotrexate administration following bortezomib treatment are as follows (1) Continue maintenance rituximab and methotrexate for six months with monthly monitoring of anti-rhGAA IgG antibody titers, (2) If antibody titers remains <12,800 while on maintenance rituximab and methotrexate then start tapering rituximab and methotrexate for another six months, and (3) if patient continues to maintain titers of <12,800 then discontinue rituximab and methotrexate and continue to monitor anti-rhGAA IgG antibody titers monthly.

Previous studies demonstrated that albuterol, a selective β2 agonist can enhance the cation independent mannose 6-phosphate receptor in skeletal muscle and further increase the efficacy of ERT in mice with Pompe disease [19]. One study showed that adding extended release (ER) albuterol in LOPD patients receiving standard dose of ERT was safe and improved forced vital capacity and 6-min walk test results compared with LOPD patients on ERT alone [20]. ER Albuterol was also demonstrated to be safe and with some beneficial effect on motor function in IOPD patients [21]. Although, the effect of ER albuterol was not studied formally in CRIM-negative IOPD patients, based on the data obtained in CRIM-positive IOPD and LOPD patients we elected to use off-label ER albuterol in our patient aiming to improve the uptake of rhGAA into skeletal muscle's lysosomes. In light of its beta agonist effect, we initiated ER albuterol only several months after beta blockers were able to be discontinued and cardiac function stabilized. Albuterol should be avoided if cardiac outflow obstruction or rhythm impairments are present. Notably, the effect of extended-release albuterol is relatively minor compared with the effects of ERT and immunomodulation and its contribution in IOPD patients needs to be further assessed in controlled clinical trials.

Emerging evidence suggests that the long term pulmonary, cardiac, and neurologic outcome of CRIM-negative IOPD patients who start treatment early (within the first month of life with ERT and immune tolerance induction) is improved compared with patients who start treatment late [22]. Accordingly, patients who are treated early (within the first 4 weeks of life) experience the best outcome with no ventilation impairment, variable degree of independent ambulation and complete oral feeding. Patients who start treatment between 4 and 15 weeks of life usually have no ventilation impairment and are able to feed orally but have variable impairment of ambulation. However, among patients who start ERT late (beyond 15 weeks of age) more than half may need some respiratory support, are not ambulatory and need G tube feeding [22]. Our patient started treatment relatively late at the age of 4.5 months. It is difficult to conclude which of all the factors in treatment and status of the patient at start of treatment played a major role but we speculate that her favorable outcome despite the late initial therapy is related both to her prematurity (corrected age of birth 2.5 months) and the aggressive treatment with high dose ERT early in the course of disease, the addition of ER albuterol and the effective immune tolerance that was achieved.

Although the current new therapies including ERT and immunomodulation strategies significantly improved the course of Pompe disease, there is still an urgent need for better and more potent therapies for Pompe disease patients especially those with the infantile onset. The ongoing research in the field includes among others, gene therapy strategies [23,24] and the emergence of next-generation ERTs that aim to improve skeletal muscle uptake of ERT by either the addition of 6 mannose phosphate residues on the molecule [25] or the use of a pharmacological chaperone which stabilizes the enzyme and improves its pharmacokinetic properties [26]. In the meantime, we describe a personalized approach aiming to better exploit more fully the current treatments.

## 4 Conclusions

The combination of early onset high dose weekly ERT, off label extended-release albuterol and successful long term immune tolerance based on monthly monitoring of anti rhGAA antibody levels provided a personalized approach that was safe and effective in our CRIM-negative IOPD patient resulting in a favorable outcome.

## Funding

This research was supported by Lysosomal Disease Network (LDN). LDN (2U54NS065768-06) is a part of the 10.13039/100006108National Center for Advancing Translational Sciences (NCATS) Rare Diseases Clinical Research Network (RDCRN). RDCRN is an initiative of the Office of Rare Diseases Research (ORDR), NCATS, funded through a collaboration between the 10.13039/100006108NCATS, the 10.13039/100000065National Institute of Neurological Disorders and Stroke (NINDS), and the 10.13039/100000062National Institute of Diabetes and Digestive and Kidney Diseases (NIDDK).

## Ethics approval

All procedures performed in the study were in accordance with the ethical standards of the institutional research committee and with the 1964 Helsinki declaration and its later amendments or comparable ethical standards. Written informed consent was obtained from a parent/guardian of the patient for participation in a Duke institutional review board (IRB)-approved study protocol (Pro00001562; Determination of Cross-Reactive Immunological Material [CRIM] Status and Longitudinal Follow-up of Individuals with Pompe disease; LDN6709 Site 206; ClinicalTrials.govNCT01665326).

## Consent for publication

Participant gave consent for publication. The data of patients included in the study are unidentified.

## Authors' contributions

Dr. Curelaru participated in the design and conceptualization of the study and led the medical writing for content.

Dr. Desai analyzed and interpreted the clinical data in particular the immunomodulatory protocol and evaluated the manuscript for content.

Dr. Fink analyzed and interpreted the cardiological data and evaluated the manuscript for content.

Dr. Zehavi analyzed and interpreted the clinical data and evaluated the manuscript for content.

Prof. Kishnani analyzed and interpreted the clinical data and the immunomodulatory protocol and the ERT protocol and evaluated the manuscript for content.

Prof. Spiegel led the composition and evaluation of the manuscript; designed and conceptualized the study and interpreted the data. Prof. Spiegel is the corresponding author for the manuscript and accepts full responsibility for the work and the conduct of the study.

## Declaration of Competing Interest

AKD has received grant support from Sanofi Genzyme and the Lysosomal Disease Network. PSK has received research/grant support from Sanofi Genzyme and Amicus Therapeutics. PSK has received consulting fees and honoraria from Sanofi Genzyme, Amicus Therapeutics, Maze Therapeutics, JCR Pharmaceutical and Asklepios Biopharmaceutical, Inc. (AskBio). PSK is a member of the Pompe and Gaucher Disease Registry Advisory Board for Sanofi Genzyme, Amicus Therapeutics, and Baebies. PSK has equity in Asklepios Biopharmaceutical, Inc. (AskBio), which is developing gene therapy for Pompe disease and Maze Therapeutics, which is developing small molecule in Pompe disease. YZ has received travel reimbursement from Sanofi Genzyme, RS has received travel reimbursement and speaker payments from Sanofi Genzyme.

## Data Availability

Data will be made available on request.
